# Gender differences in the effects of urban environment on nighttime exercise behaviours: a qualitative study

**DOI:** 10.3389/fpsyg.2024.1465737

**Published:** 2024-11-13

**Authors:** Yuqin Su, Xiaoli Pan, Yike Li, Guanchong Li, Guodong Zhang

**Affiliations:** ^1^College of Physical Education, Institute of Sport Science, Southwest University, Chongqing, China; ^2^International College, Krirk University, Bangkok, Thailand

**Keywords:** urban environment, nighttime exercise, behaviours, gender, qualitative study

## Abstract

**Objective:**

With the acceleration of urbanization, nighttime exercise behaviours have rapidly emerged. Existing research indicates a correlation between urban environments and physical activity; however, studies focusing specifically on nighttime are still insufficient, particularly regarding gender differences. This study aims to identify the key factors in urban environments that influence residents’ nighttime exercise behaviours and to explore the gender differences within these influences.

**Methods:**

Purposeful sampling was employed to conduct semi-structured interviews with 30 residents who regularly engage in nighttime exercise. All transcribed interviews were analyzed using Colaizzi’s phenomenological data analysis method.

**Results:**

The findings revealed that physical environment and environmental perception are the two primary factors influencing nighttime exercise behaviour. These factors are further divided into 10 specific sub-themes: lighting, green spaces, site facilities and layout, traffic coherence, entertainment facilities, smart sports equipment, sense of safety, convenience, pleasure, and sense of belonging.

**Conclusion:**

Females demonstrate a heightened sensitivity to the perception of the physical environment, placing greater emphasis on the feelings and experiences it provides. Males, on the other hand, focus more on the direct impact of the physical environment, such as its specific effects on exercise performance. Future urban planning and public policy should give greater consideration to gender differences in the use of urban exercise facilities, ensuring that nighttime exercise environments meet the needs of residents of different genders. This approach will contribute to enhancing overall community vitality and improving residents’ health.

## 1 Introduction

Urbanization is one of the prominent features of contemporary societal development. According to data from the United Nations, about 56% of the global population resided in urban areas in 2020, with this proportion expected to rise to 68% by 2050 (WHO, 2021). Urbanization not only drastically alters economic structures and social layouts but also profoundly impacts the living environments and lifestyles of urban residents. With rapid urban expansion comes a series of intensified environmental and social issues, such as the reduction of green spaces (Li et al., 2019), deterioration of air quality (Angel et al., 2011), increase in noise pollution (King et al., 2012), and densification of living spaces (Arfanuzzaman and Dahiya, 2019). These issues not only attract widespread attention but are also seen as key factors affecting the quality of daily life and physical health of urban dwellers. Studies have shown that with the proliferation of private cars, traffic congestion has become increasingly severe, along with rising levels of air and noise pollution, which are significantly associated with the incidence of respiratory and cardiovascular diseases (Li et al., 2009; Pizzi et al., 2016; Mueller et al., 2017; Münzel et al., 2022). Furthermore, the constriction of urban spaces and the lack of public facilities limit the frequency of physical exercise among residents, further threatening their health status (Stevenson et al., 2016; Giles-Corti et al., 2022). In response to these challenges, the World Health Organization launched the “Healthy Cities” initiative, aimed at optimizing urban design to improve residents’ living environments, thereby enhancing public health levels (Flynn, 1996; Kenzer, 1999). This movement emphasizes integrating public health perspectives into urban planning and policy-making to achieve a balance between environmental sustainability and resident health.

Insufficient physical activity is another significant issue in the urbanization process, especially in more developed regions such as Europe, America, and parts of East Asia (Guthold et al., 2018, 2020; Strain et al., 2024). This issue was particularly emphasized by WHO (2024). Numerous studies have demonstrated a close connection between urban environments and the level of physical activity among residents; the urban setting can either facilitate or hinder physical activities (Handy et al., 2002; Chandrabose et al., 2021). These findings, primarily derived from cross-sectional surveys, provide initial clues for understanding and addressing the issue. Urban transportation strategies and the configuration of public spaces directly impact residents’ exercise habits. Cases from various countries show that having well-maintained bicycle and walking paths, along with increased greenery and maintaining neighbourhood cleanliness and safety, can significantly boost the frequency of daily walking and cycling among residents (Toftager et al., 2011; Lu et al., 2018; Yang et al., 2021). Additionally, the accessibility and safety of a city are considered key factors influencing residents’ decisions to engage in physical activities outdoors. Ecological models offer a framework to understand these impacts, emphasizing that behavioural changes undergo influences at multiple levels, where positive support from the external environment is crucial (Hoehner et al., 2005; Sallis et al., 2012). Good lighting and a pleasant neighbourhood environment have been proven to correlate with higher levels of physical activity among residents (Ding et al., 2011; Campbell and Janssen, 2022). This indicates that improving infrastructure and enhancing the usability and safety of urban spaces can effectively promote physical activity.

Understanding how gender influences participation in physical activities within urban environments is crucial, as significant differences exist between genders in patterns and levels of physical activity participation (Johnson et al., 2009; Cochrane Methods Group, 2020). A systematic review covering 36 studies indicates clear distinctions in how the built environment affects physical activity between men and women (Tcymbal et al., 2020). Numerous studies have shown that women generally have lower levels of physical activity compared to men, and their perceptions of the environment differ, which may be a significant factor limiting their participation (Ranasinghe et al., 2013; Mielke et al., 2017; Guthold et al., 2018). The theory of environmental perception provides a framework for understanding these gender differences (Momsen, 2000; Garcia Bengoechea et al., 2005). According to this theory, an individual’s perception of their environment directly influences their behaviours, including the frequency and type of physical activity (Derose et al., 2019). Women may pay more attention to safety and social dimensions in the environment, which play crucial roles in their decision-making about engaging in outdoor activities (Foster and Giles-Corti, 2008). Studies indicate that when women perceive an area as safe and conducive to relaxation, their likelihood of participating in outdoor activities increases (Bennett et al., 2007; Adlakha et al., 2016). Additionally, gender roles and cultural expectations greatly shape the behavioural patterns of men and women. In many cultures, women may avoid engaging in physical activities in certain public spaces due to concerns about safety or non-conformity to societal expectations (Reis et al., 2009).

Nighttime exercise is becoming increasingly favoured by urban residents as an exercise time slot, especially for office workers who are pressed for time during the day (Su et al., 2024). Often, this is the only feasible opportunity for them to exercise. Due to the uniqueness of the nighttime, the impact of various urban environmental factors on exercise behaviour is more pronounced. Additionally, gender factors become particularly significant in the context of nighttime exercise, as men and women may perceive and react to the nighttime environment differently. Although existing research has explored the impact of the urban built environment or environmental perceptions on residents’ physical activity through cross-sectional surveys, some studies have also attempted to examine gender differences in the urban environment from a quantitative perspective (Boone-Heinonen et al., 2010; McCormack and Shiell, 2011; Feuillet et al., 2016; Kärmeniemi et al., 2018). Despite the growing interest in how urban environments influence physical activity, there remains a significant gap in understanding the specific dynamics of nighttime exercise and the role of gender in these contexts. Previous research predominantly focuses on daytime activities and offers limited qualitative insights into how urban settings impact nighttime exercise behaviours. This study aims to fill this gap by investigating how urban environmental factors, particularly lighting and safety perceptions, influence nighttime exercise among men and women in urban settings. This not only enhances our understanding of how the nighttime urban environment shapes physical activity patterns but also provides a new perspective considering gender differences, offering practical suggestions for urban planning.

## 2 Methods

### 2.1 Study design

We employed a semi-structured interview approach to gain an in-depth understanding of participants’ experiences with nighttime exercise in urban settings. Participants were selected through purposive sampling from Chongqing central urban areas, focusing on those who engaged in nighttime exercise at least two to three times per week. A balanced sample of 15 males and 15 females was recruited to examine gender differences. The interview guide was developed from literature reviews and expert consultations, followed by pilot testing to refine the questions. Face-to-face interviews were conducted near participants’ exercise locations, ensuring contextual relevance. Data analysis employed Colaizzi’s phenomenological method to systematically identify key themes from the participants’ experiences.

### 2.2 Participants

The study was conducted in Chongqing, one of China’s four direct-controlled municipalities. We selected residents from the central urban areas of Chongqing using a purposive sampling strategy, a selective sampling technique specifically utilized for qualitative research (Suri, 2011). Purposive sampling aimed to recruit participants who regularly engage in nighttime exercise, ensuring the relevance and depth of the data collected. Eligibility criteria for recruitment included: residents living in the central urban areas who engage in nighttime exercise at least two to three times per week (Haskell et al., 2007) and are willing to express their thoughts candidly. To recruit potential participants, research booklets were extensively distributed in public spaces across all urban districts. Additionally, participant recruitment was conducted on a rolling basis to ensure a balanced representation of key sociodemographic variables such as age, marital status, educational background, and gender. The study recruited 15 males and 15 females to facilitate a comprehensive comparison and analysis of gender differences in nighttime exercise behaviours.

### 2.3 Data collection

In this study, the research team initially developed a semi-structured interview guide by thoroughly reviewing relevant literature and engaging in discussions with domain experts. To ensure the effectiveness of the guide, a pilot test (*n* = 2) was conducted. Based on the feedback received, adjustments were made, and the final interview guide was established (Table 1). The formal interviews were conducted face-to-face, on a one-on-one basis, near the participants’ regular nighttime exercise locations by members of the research team. Before starting the interview, researchers clearly explained the study’s objectives, methodology, and the purpose of using recording devices, while also promising to strictly protect participants’ personal information to ensure their privacy. Interviews typically lasted between 30 and 45 min. Participants were compensated with 50 RMB (approximately 7 USD) after the interview. Research team members were responsible for transcribing the recorded content verbatim into text and anonymizing the transcripts before providing them to participants to verify and confirm the accuracy of the interview content.

**Table 1:** Interview outline.

1. What time do you usually exercise and how long does each session last on average?
2. Please describe the environment of your chosen exercise location, including details you consider important.
3. What factors influence your exercise experience? How do they specifically affect it?
4. What factors do you typically consider when choosing the time and place for nighttime exercise?

### 2.4 Data analysis

Two researchers (Y Q.S and X L.P) employed Colaizzi’s phenomenological method to analyze all transcribed interview data (Elo and Kyngäs, 2008). To ensure consistency and reliability in the analysis, a dual-coding approach was utilized. Initially, both researchers independently coded a subset of the data to identify key themes and concepts. Following this, they convened to compare their coding results and discuss any discrepancies. To assess intra- and inter-observer reliability, we calculated the Kappa coefficient for the coding consistency between the two researchers. A Kappa value of 0.80 or higher was deemed acceptable, indicating strong agreement. The results showed a Kappa coefficient of 0.87, suggesting a high level of reliability in the coding process. The researchers then continued with the thematic analysis, identifying sub-themes and main themes, ensuring that all relevant experiences were thoroughly explored and reached saturation. The findings were subsequently validated by revisiting participants to confirm that the results accurately reflected their experiences.

### 2.5 Ethics statement

This study adheres to the ethical standards set forth in the Declaration of Helsinki. The research was approved by the Ethics Committee of the School of Physical Education at Southwest University, with approval number (SWU-PE-20240524). Participants were briefly informed about the purpose of the study during recruitment. Written informed consent was distributed and collected from participants who agreed to participate in the interviews. Additionally, permission to record the interviews was obtained from the participants prior to conducting them.

## 3 Results

### 3.1 Study participants

The study included 30 participants, comprising an equal gender ratio of 15 females and 15 males. The average age of the participants was 37 years. Regarding educational background, 50% of the interviewees possessed a bachelor’s degree. In terms of marital status, approximately 56.7% of the participants were married (see Table 2).

**Table 2:** Basic information of participants.

Variable	Category	Mean (SD)	Percentage (*N*)
Gender	Male		50% (15)
	Female		50% (15)
Age (year)		36.7 ± 10.8(13.8)	
Educational status	Junior high school		10% (3)
	Senior high school		20% (6)
	Undergraduate		50% (15)
	Postgraduate		20% (6)
Marital status	Unmarried		33.3% (10)
	Married		56.7% (17)
	Divorce		10% (3)

### 3.2 Qualitative findings

After organizing and analyzing the interview data, we identified 2 main themes and 10 sub-themes.

#### 3.2.1 Physical environment

The urban physical environment plays a decisive role in influencing residents’ outdoor nighttime exercise behaviours, a finding consistently confirmed across interviews and evident among both males and females. Several aspects of the physical environment, such as the layout and brightness of street lighting, the availability and maintenance of sports facilities, and the comprehensiveness of public safety measures, directly impact individuals’ choices regarding when to exercise and how frequently they engage in such activities.

##### 3.2.1.1 Sub-theme 1: lighting

Up to 90% of participants, when asked about the main factors influencing their decisions to exercise at night, initially cited the lighting conditions of the exercise locations, highlighting the crucial role of adequate lighting in facilitating nighttime outdoor activities. Particularly among female participants, there was a heightened sensitivity to the intensity and arrangement of lighting fixtures. Many expressed that insufficient public lighting could make otherwise preferred exercise locations unviable due to feelings of insecurity.

*"I feel that the brightness of the lights at the exercise location greatly affects me. I usually walk along Binjiang Road on Wednesdays and Thursdays, and on Fridays, I go to the Chengnan Sports Ground. I clearly noticed that when the lighting is good on Fridays, I walk twice as long compared to Binjiang Road. "*

*"Because I have some astigmatism, I need to see the lights clearly, or else I won't come here. In places that are too dark at night, and because sometimes I can't see clearly due to my astigmatism, I might get hurt, so I choose well-lit places. "*

*"Previously, I walked under a bridge, circling around the nearby street park. There was a stretch where ground lights would shine directly into my eyes, which was very uncomfortable. I really dislike walking that segment, but I can't avoid it."*

Men also displayed a strong concern for lighting conditions during nighttime exercise, especially for sports like basketball and badminton that require high levels of illumination. Male participants commonly emphasized the importance of adequate lighting brightness on courts to meet the demands of high-intensity sports. Additionally, the timing for lights being turned off at outdoor sports facilities was a significant concern for men, as extending lighting hours could effectively increase their opportunities to engage in nighttime exercise.

*"The most important thing for us when playing basketball is the lighting on the court. You see, so many people prefer to queue up here rather than go to the adjacent court. No one wants to play on the adjacent court; you can't even see clearly when shooting.*

*"My job inevitably involves overtime. After work, when I finally have some time to myself and come here to exercise, the lights go out after just a short while. It's dark, and I can't see anything, which makes me worry about getting injured, so I just have to go home. It would be great if the lighting hours could be extended, but I'm not sure which department to contact about this."*

##### 3.2.1.2 Sub-theme 2: green spaces

The level of greenery and vegetation in exercise environments was mentioned by a majority of the interviewees, with female participants showing particularly high interest. Only two male respondents believed that green spaces influence their nighttime exercise behaviours. Many women stated that abundant green vegetation makes them feel the freshness of the air and improves their mood, viewing proximity to nature after a busy workday as a way to relieve stress and escape the complexities of urban life. However, a few also raised concerns that dense vegetation at night might conceal potential risks, such as hard-to-see obstacles or small animals, which could pose safety hazards.

*"I just love walking on the big lawn in the park; the fresh scent of the grass makes me feel comfortable, especially in the early summer."*

*"Places with lots of greenery make me feel like I'm briefly escaping from the noisy city and complex work. It's one of the few opportunities I get to clear my mind."*

*"Young people, you know, have rich imaginations. The small park below my house has a large area covered in greenery. When I used to run there, I always felt like snakes or rats might suddenly appear, so I switched to running here now."*

##### 3.2.1.3 Sub-theme 3: site layout and facilities

The rational design of site layout and facilities significantly impacts nighttime exercise behaviours in urban physical environments. Proper spatial distribution not only optimizes the utilization of spaces but also enhances their attractiveness to those exercising at night. Men and women differ in their concerns about site layout; male participants involved in competitive sports like basketball or football worry about children accidentally entering the play area, which could disrupt the game or increase the risk of injuries. Women, on the other hand, express concerns about being bumped into by swiftly moving children while engaging in rhythmic exercises such as jogging or aerobics. Participants also commonly reported issues with the maintenance of outdoor public sports facilities, noting that inadequate upkeep, such as damaged equipment not being repaired promptly, forces residents to exercise in suboptimal conditions. This not only dampens enthusiasm for exercising but also reduces the frequency of exercise sessions.

*"Because the place where I exercise is a comprehensive area, many kids also come to play with their classmates. There was an incident where a kid picking up a balloon was knocked over by boys playing ball. Whose fault is that? It's hard to say, and such incidents are unavoidable."*

*"Look at that ping-pong table, it's rusted, and no one seems to care. The net is also missing a few threads, only us older folks come here for a bit of entertainment. Of course, we come because it's close to home; who wouldn't prefer better facilities?"*

*"We dance in the square here every evening; it has become a habit. If we don't come, it must be for a special reason. One of my neighbours was knocked down here by some kids racing their bikes, and she had to rest for two months."*

##### 3.2.1.4 Sub-theme 4: traffic connectivity

Traffic connectivity significantly impacts residents’ choices of nighttime exercise locations and their accessibility. Approximately 80% of the interviewees preferred exercise locations within 3 km of their residence, emphasizing the importance of proximity. Both men and women indicated that busy roads are a barrier to participating in nighttime exercise, restricting their willingness to engage in outdoor activities. Additionally, participants who rely on cycling for exercise expressed particular concern about traffic safety, especially in areas lacking dedicated bicycle lanes. They consider good traffic connectivity and a safe cycling environment to be crucial for nighttime exercise.

*"I usually choose to run in a park near my home because I'm very familiar with the routes. If the route requires crossing busy traffic areas, I'll avoid exercising there. I've tried it a few times in the evening, but it felt very unsafe, especially when crossing those major roads with lots of vehicles—I worry about accidents."*

*"I like to cycle to a slightly farther sports ground because the facilities there are newer. In our area, there's a lack of clear bike lanes, especially for cycling at night when there are many vehicles. The absence of dedicated bike lanes feels very dangerous, which sometimes makes me consider changing the location or just not going at all."*

*"Being able to get there in a few minutes by walking is very convenient for me. The nearby roads are also good to walk on, and the road planning along the way is very clear."*

##### 3.2.1.5 Sub-theme 5: entertainment facilities

While discussing the role of entertainment facilities in the urban physical environment, it was noted that although mentioned by fewer interviewees, their importance is significant, especially for families with children. These participants highlighted the need for multifunctional entertainment facilities that not only accommodate adult exercise routines but also provide spaces for children’s physical activities. Such facilities encourage family involvement, foster interaction, and help keep children active, supporting their healthy development.

*"As a father of two, I really hope for more family-friendly recreational facilities. The children's area in the park we often go to is quite small and gets very crowded. If there was more space, the kids could move around more freely, and I could jog or do some stretching exercises nearby, benefiting the whole family."*

*"I've noticed that when I take my kids to places with sports facilities, we tend to stay longer, and the overall activity time for the family increases."*

*"In our community, there's a lack of sports facilities suitable for the whole family to use together. Most of the facilities are either designed for adults, like gym equipment, or for small children, like slides and swings."*

##### 3.2.1.6 Sub-theme 6: smart sports equipment

Male participants in this study showed particular interest and anticipation for smart sports equipment. They emphasized the potential value of such devices during nighttime exercise, especially the capability to provide real-time display of exercise data, such as feedback on distance, speed, heart rate, and other key metrics. This technological integration not only increases the interactivity and motivation of the exercise but also helps athletes more effectively monitor and adjust their workout intensity. Additionally, some interviewees suggested that these smart devices should include emergency call functions to enhance safety during nighttime exercise.

*"Smart sports facilities are great. If the fitness equipment in parks could display exercise data for users and provide some fitness guidance, it would really attract people to participate in exercise. The integration of such technology can truly change the way we exercise."*

*"Last time I visited another city, I saw many emergency aid devices around the sports grounds, but I haven't seen any around here. Having some would definitely be better, especially since there are many elderly people in this area."*

#### 3.2.2 Perceived environment

Perceived environment typically refers to an individual’s subjective evaluation and feelings about various factors in their surrounding environment. This perception significantly influences participants’ psychology and sentiments related to engaging in nighttime exercise. Perceptions of the environment are influenced not only by external factors but also by personal experiences and emotional states.

##### 3.2.2.1 Sub-theme 1: sense of safety

In this study, the sense of safety is a frequently mentioned theme in the perception of the nighttime exercise environment. Female participants particularly emphasized that a sense of security during outdoor nighttime exercise is crucial for them. Feelings of insecurity or potential dangers can quickly lead them to discontinue their exercise and might impact their long-term enthusiasm for physical activity. Women’s sense of safety is primarily influenced by factors such as pedestrian traffic, lighting, and familiarity with the environment. In contrast, while men also focus on safety, they tend to be more concerned with the physical conditions of the exercise location, such as facility maintenance and the age of equipment, and the sports injuries these factors might cause. Additionally, men who cycle also pay attention to traffic safety.

*"Once, the streetlights in the park garden were out for a few days, and I felt very scared while running. Even though I've walked that path many times during the day, I still felt very unsafe. I found myself unconsciously speeding up as if trying to escape."*

*"In the coldest part of winter, there are very few people around here, and I feel very unsafe. The potential dangers make me afraid."*

*"The exercise location I choose must have complete facilities and be well-maintained. For example, at the basketball court, if the hoop or floor is damaged, it's very easy to fall or sustain other sports injuries. This is a safety issue I am very concerned about."*

*"Safety standards of exercise facilities are one of the factors I consider when choosing a place to exercise. I pay special attention to whether the ground is even because I am worried about getting injured during physical activities."*

##### 3.2.2.2 Sub-theme 2: convenience

The results from participants indicate that convenience is one of the key factors influencing nighttime exercise participation, particularly reflected in the accessibility of exercise locations. Generally, the accessibility of a location, especially its walkability, is a crucial factor that increases its frequency of use. Exercise venues within walking distance tend to attract more exercisers, and most respondents, both male and female, prefer to choose exercise locations within a 3 km radius of their homes. This preference not only reflects a high regard for time and transportation convenience but also indicates that, amidst the busyness of daily life and the precious nature of nighttime, convenient exercise options become a prioritized consideration.

*"I have a great running track near my home, just about a five-minute walk away. This really motivates me to go out and run every evening. If it weren't for this track, I might just choose to stay at home and do other things."*

*"Since I'm very busy during the day, having an exercise location that I can quickly reach in the evening is also very important to me."*

*"I get home from work late, so I go running downstairs. I don't want to go far; I don't have much time."*

##### 3.2.2.3 Sub-theme 3: enjoyment

The enjoyment derived from physical activity significantly influences some participants’ decisions about when and how often to exercise. There are notable gender differences in how participants perceive and respond to their exercise environment. Women generally are more sensitive to environmental aesthetics and emphasize that well-maintained green spaces, beautifully trimmed gardens, and warm lighting can greatly enhance their exercise enjoyment and mood. Men, however, tend to derive pleasure more from their personal performance in sports and the functionality of sports facilities, such as the performance on a basketball court, the adequacy of lighting, and the condition of the equipment. These elements significantly impact their level of enjoyment during exercise.

*"I really like the warm lighting; exercising under dim, yellow lights at night makes me feel relaxed."*

*"The park is specially decorated for certain holidays, and seeing those designs makes my exercise sessions very enjoyable."*

*"I pay close attention to my performance on the basketball court. Playing in areas with smooth surfaces and good lighting, I feel like I perform very well, which keeps me happy all evening."*

*"Bright lights make me feel excited."*

##### 3.2.2.4 Sub-theme 4: sense of belonging

A sense of belonging plays a critical role in motivating individuals to regularly participate in nighttime exercise, directly influencing their emotional engagement and involvement with the exercise environment. Particularly among some older women, this sense of belonging is closely linked to the social environment of their community. These women prefer to exercise within their neighbourhoods not only because of the convenience of location but also because it allows them to meet and exercise alongside neighbours and acquaintances. This social interaction enhances their sense of security and belonging, encouraging them to participate more frequently in exercise activities. In contrast, young men often mention a sense of belonging; they prefer to exercise in specific areas where their friends and classmates are also active, as the presence of peers significantly boosts their sense of belonging.

*"I walk in my neighbourhood. Proximity is definitely one reason, but also because I can walk with my neighbours. As homeowners, we feel a sense of belonging here."*

*"My classmates all live nearby, and we always arrange to play basketball together. Familiar places and familiar friends really give a sense of belonging. We always form teams together, like a real team."*

## 4 Discussion

This study employs qualitative research methods, conducting semi-structured interviews with 30 urban residents to explore how the urban environment influences their nighttime exercise behaviours, with a particular focus on the differential impacts of gender. The research identified two main themes—physical environment and perceived environment—and further refined these into 10 sub-themes: lighting, green spaces, site facilities and layout, traffic coherence, entertainment facilities, smart sports equipment, sense of safety, convenience, pleasure, and sense of belonging.

Our findings indicate that, during nighttime, the data related to lighting emerged as the most significant theme, highlighting a universal recognition among both men and women of its importance. This aligns with previous research, which suggests that a high-quality lighting environment can significantly promote residents’ nighttime exercise behaviours. Specifically, it has been shown that individuals in areas with better illumination tend to walk longer distances (Kim et al., 2014; Portnov et al., 2020). This phenomenon may stem from higher lighting brightness being able to suppress the secretion of melatonin, thereby reducing sleepiness and enhancing energy levels, which increases individuals’ inclination to be active (De Toledo et al., 2023; Lu et al., 2023). Additionally, from the perspective of behavioural mechanisms, good lighting not only improves the visual clarity of the environment, reducing the potential risks of nighttime activities (Rahm et al., 2020; Svechkina et al., 2020), but also increases the nighttime usability of public spaces, thereby motivating residents to engage in more frequent physical activities. Furthermore, we found differences in lighting preferences between genders. Female participants tend to pay more attention to the colour temperature of lighting, generally believing that lights with a white colour temperature create a more comfortable environment, thereby being more willing to increase the frequency of physical activities. This finding is consistent with previous research, which indicates that white light can enhance the brightness of an area and thus improve individuals’ psychological sense of security (Knight, 2010; Peña-García et al., 2015). In contrast, males emphasize the intensity of lighting more, believing that high-intensity lighting helps provide clear visibility, and are more concerned about whether lighting facilities can continuously provide stable and long-duration illumination to support sustained activities at night.

Green exercise has been a hot topic of interest in the academic community in recent years. Our study confirms from a subjective perspective that there is an association between the coverage of green vegetation and more active exercise behaviours, particularly noting that women place greater importance on green environments compared to men (Sander et al., 2017; Sillman et al., 2022). This phenomenon can be explained by the Attention Restoration Theory, which suggests that green environments provide individuals with opportunities to escape urban stress and the daily burdens of work (Ratcliffe, 2021; Yao et al., 2021). Additionally, elements of the natural environment, such as grass and leaves swaying in the wind and flowers in parks, often possess significant aesthetic value, which may particularly attract the attention of women. Further analysis indicates that gender differences in the preference for green exercise may stem from women’s greater sensitivity to natural beauty (Chen et al., 2015; Hoyle et al., 2017), consistent with psychological research that suggests women generally display higher sensitivity in emotional resonance and aesthetic experiences (Bloise and Johnson, 2007; Poláčková Šolcová and Lačev, 2017).

The layout of facilities and the coherence of traffic are two key aspects universally valued by the respondents. Research findings suggest that gender differences in these areas are relatively small. Respondents commonly believe that reasonable planning of the site is crucial for ensuring effective exercise outcomes and the continuity of exercise routines. This finding emphasizes the core role of orderly and logical site layouts in promoting residents’ daily exercise activities (Frank et al., 2005; Mooney et al., 2020). Moreover, feedback about transportation highlights the importance of pedestrian and bicycle paths in urban planning. Effective traffic design involves not only the efficiency of vehicular traffic but also providing safe and convenient routes for walking and cycling (Merom et al., 2003; Pikora et al., 2003; Van Holle et al., 2012). This is particularly key for encouraging physical activity in urban settings. Our study further confirms from a side perspective that residents tend to choose exercise locations within a 3 km radius of their homes, underscoring the importance of the quality of the neighbourhood environment in promoting physical activity (Humpel et al., 2004; Cerin et al., 2007). Surprisingly, the attention to entertainment facilities showed unexpected gender differences, with males showing more concern, differing from previous research findings (Mackenbach et al., 2018; Smith et al., 2019). Men not only expect these venues to meet their daily exercise needs but also hope they support family-oriented activity participation, thereby promoting interaction among family members and sharing a healthy lifestyle. Consistent with previous research (Song et al., 2017; Kim et al., 2019), only men showed a particular expectation for smart sports equipment, hoping for more high-tech products in the urban exercise environment to facilitate real-time monitoring and optimization of their workout performance.

While the physical environment provides the foundational conditions for nighttime exercise, it is ultimately the individual’s perception of these features that determines their behavioural responses. Under the theme of environmental perception, there are clear gender differences in perceptions of safety and pleasure. The perception of safety has a very significant impact on women’s participation in nighttime exercise, as evidenced by studies in multiple countries around the world (Bennett et al., 2007; Bamana et al., 2008; Dias et al., 2019). Research indicates that women have a higher sensitivity to safety in the environment, which not only affects their daily behaviour patterns but also becomes a decisive factor when deciding whether to participate in nighttime exercise (Fotios et al., 2015). The roots of gender differences in safety perception can be explored through theories of social psychology and gender roles. Social psychological studies suggest that women are generally more concerned about potential social threats than men (Börjesson, 2012; Loukaitou-Sideris, 2014), which may be related to biological differences and gender roles formed during the socialization process. Pleasure also shows significant gender differences; studies have shown that women, compared to men, are more inclined to derive happiness from their environment and use it to improve their mood (Nurse et al., 2010). Women have higher aesthetic and emotional needs towards the environment, which leads them to pay more attention to the aesthetics and comfort of the environment, such as the beauty of green spaces and the cleanliness and safety of venues. These factors directly affect their sense of pleasure. Conversely, men’s sense of pleasure often stems more from their performance in sports activities that evening, such as the intensity of the exercise and goals achieved, which relates to their typical high regard for achievement and competition. From a psychological perspective, women are more likely to use the external environment to regulate their emotional states, a viewpoint supported by previous research (Ball et al., 2001). Men, on the other hand, tend to derive satisfaction and pleasure from completing specific tasks and demonstrating personal capabilities (Hayashi, 1996; Gerdin, 2017).

## 5 Limitations

In this study, we must acknowledge certain limitations. Methodologically, this research employs qualitative methods to explore the potential impacts of the urban environment on nighttime exercise behaviours. Although qualitative methods provide deep insights and an understanding of individual experiences, the reliance on data primarily based on participants’ subjective narratives limits our ability to infer strong causal relationships from the data. Secondly, regarding sample selection, this study’s subjects were confined to Chongqing. While this aids in understanding the specifics of a particular area, such geographical limitation might result in findings that do not fully represent other regions or cultural contexts. Therefore, the generalizability and extrapolation of the study findings are somewhat restricted. Furthermore, we did not systematically record the specific types of exercise participants engaged in, which limits our ability to compare the effects of environmental factors like lighting across different types of exercise and genders. Lastly, despite these limitations, we believe that the findings of this study hold significant value for guiding future related research. In future studies, we plan to expand the sample range to include more regions and participants from diverse backgrounds to enhance the representativeness and universality of the research. Additionally, we consider using quantitative methods to supplement the qualitative analysis to strengthen the reliability of causal inferences and further validate and deepen the findings of this study.

## 6 Conclusion

In this qualitative study, we explored the impact of the urban environment on nighttime exercise behaviours through semi-structured interviews with 30 respondents. The results reveal that the physical environment and environmental perception are two major influencing factors of nighttime exercise behaviours, further divided into 10 specific sub-themes: lighting, green spaces, site facilities and layout, traffic coherence, entertainment facilities, smart sports equipment, sense of safety, convenience, pleasure, and sense of belonging. Significant gender differences were observed: women are more sensitive to the environment’s aesthetics and safety, whereas men focus on its direct impact on physical performance. Future urban planning should consider these differences to enhance exercise facilities for both genders, with a specific focus on improving lighting, layout, and safety features to accommodate and encourage nighttime exercise among residents, thus boosting community vitality and health.

## Data Availability

The raw data supporting the conclusions of this article will be made available by the authors, without undue reservation.
